# Association between six-minute walk distance and long-term outcomes in patients with pulmonary arterial hypertension: Data from the randomized SERAPHIN trial

**DOI:** 10.1371/journal.pone.0193226

**Published:** 2018-03-28

**Authors:** Rogério Souza, Richard N. Channick, Marion Delcroix, Nazzareno Galiè, Hossein-Ardeschir Ghofrani, Pavel Jansa, Franck-Olivier Le Brun, Sanjay Mehta, Loïc Perchenet, Tomás Pulido, B. K. S. Sastry, Olivier Sitbon, Adam Torbicki, Lewis J. Rubin, Gérald Simonneau

**Affiliations:** 1 Pulmonary Department, Heart Institute, University of São Paulo Medical School, São Paulo, Brazil; 2 Pulmonary and Critical Care, Massachusetts General Hospital, Boston, Massachusetts, United States of America; 3 Department of Pneumology, Gasthuisberg University Hospital, Leuven, Belgium; 4 Department of Experimental, Diagnostic and Specialty Medicine-DIMES, Bologna University Hospital, Bologna, Italy; 5 Medical Clinic II/V, University of Giessen and Marburg Lung Center (UGMLC), Giessen, Germany; 6 Department of Medicine, Imperial College London, London, United Kingdom; 7 Clinical Department of Cardiology and Angiology, 1st Faculty of Medicine, 2nd Medical Department, Charles University, Prague, Czech Republic; 8 Department of Biostatistics, Actelion Pharmaceuticals Ltd, Allschwil, Switzerland; 9 Department of Medicine, Division of Respirology, London Health Sciences Centre – Victoria Hospital, Western University, London, Ontario, Canada; 10 Global Medical Affairs, Actelion Pharmaceuticals Ltd, Allschwil, Switzerland; 11 Cardiopulmonary Department, Ignacio Chávez National Heart Institute, Mexico City, Mexico; 12 Department of Cardiology, CARE Hospitals, Hyderabad, India; 13 Assistance Publique–Hôpitaux de Paris, Service de Pneumologie, Hôpital Bicêtre, Le Kremlin-Bicêtre, France; 14 Université Paris-Sud, Laboratoire d’Excellence en Recherche sur le Médicament et L’innovation Thérapeutique, Le Kremlin-Bicêtre, France; 15 INSERM U-999, Centre Chirurgical Marie-Lannelongue, Le Plessis-Robinson, France; 16 Department of Pulmonary Circulation and Thromboembolic Diseases, Medical Center of Postgraduate Education, Otwock, Poland; 17 University of California, San Diego School of Medicine, La Jolla, California, United States of America; Kurume University School of Medicine, JAPAN

## Abstract

**Background:**

Patients with pulmonary arterial hypertension who achieve a six-minute walk distance of 380–440 m may have improved prognosis. Using the randomized controlled trial of macitentan in pulmonary arterial hypertension (SERAPHIN), the association between six-minute walk distance and long-term outcomes was explored.

**Methods:**

Patients with six-minute walk distance data at Month 6 were dichotomized as above or below the median six-minute walk distance (400 m) and assessed for future risk of pulmonary arterial hypertension-related death or hospitalization and all-cause death. Additionally, six-minute walk distance values at baseline, Month 6 and the change from baseline to Month 6 were categorized by quartiles. All associations were analyzed by the Kaplan–Meier method using a log-rank test and Cox regression models.

**Results:**

Patients with a six-minute walk distance >400 m vs. ≤400 m at Month 6 have a reduced risk of pulmonary arterial hypertension-related death or hospitalization (hazard ratio 0.48; 95% confidence interval 0.33–0.69). The risk was also lower for patients with higher quartiles of six-minute walk distance at baseline or Month 6 (baseline: hazard ratio [Q4 (>430 m) vs. Q1 (≤300 m)] 0.23; 95% confidence interval 0.15–0.36; Month 6: hazard ratio [Q4 (>455 m) vs. Q1 (≤348 m)] 0.33; 95% confidence interval 0.19–0.55). In contrast, six-minute walk distance changes at Month 6 were not associated with the risk of pulmonary arterial hypertension-related death or hospitalization (*p* = 0.477). These findings were consistent when adjusted for known confounders. Similar results were observed for the risk of all-cause death up to end of study.

**Conclusions:**

Patients with pulmonary arterial hypertension walking >400 m had better long-term prognosis. Although changes in six-minute walk distance were not associated with long-term outcomes, assessing absolute six-minute walk distance values remains important in the clinical management of patients with pulmonary arterial hypertension.

## Introduction

Pulmonary arterial hypertension (PAH) is a relentlessly progressive disease characterized by an increase in pulmonary artery pressure and pulmonary vascular resistance, leading to right ventricular failure and poor survival [1, 2]. As a consequence of the pulmonary hemodynamic abnormalities, there is also a progressive decline in the functional and exercise capacity of patients with PAH.

During recent decades, an important improvement in the management of PAH has been witnessed through the development of targeted therapies that have a significant impact on hemodynamics, exercise capacity and survival [3]. The efficacy of these therapies has been assessed mainly in clinical trials by the short-term (3–6 months) improvement of exercise capacity, measured by six-minute walk distance (6MWD) [3].

The 6MWD test is a simple and reproducible measure of exercise capacity, and, although representing a submaximal level of functional capacity for most patients, has a close association with a maximal cardiopulmonary exercise test [4]. Data from observational studies and randomized trials suggest that absolute values of 6MWD are associated with better prognosis [5–7], whereas short-term changes in 6MWD are not [6–10]. This is reflected in international guidelines that describe treatment goals in PAH, in which a 6MWD value of greater than 380 m to 440 m is suggested to indicate response to therapy or better prognosis [11, 12].

Six-minute walking distance thresholds are commonly used in conjunction with other clinical, functional and hemodynamic parameters when assessing and treating patients with PAH. Several studies, using parameters outlined in the ESC/ERS guidelines, have shown that patients at low risk have a better prognosis than patients stratified at high risk [13, 14]. Similarly, a recent study in Japan demonstrated significantly better prognosis in patients with 6MWD >372 m, mean pulmonary arterial pressure ≤46 mmHg and cardiac index >2.5 L/min/m^2^ [15].

The SERAPHIN study, with a median treatment duration of 2.2 years, was the first long-term event-driven outcome trial in PAH [16]. As 6MWD was measured at baseline and at Month 6, SERAPHIN provides a unique opportunity to analyze the association between 6MWD and long-term clinical outcomes in PAH.

## Methods

### Study design and patients

SERAPHIN was a multicenter, randomized controlled, event-driven study designed to assess the long-term efficacy and safety of macitentan in patients with PAH (NCT00660179). The study design has been described in detail elsewhere [17]. Briefly, patients were included if they were 12 years of age or older and diagnosed by right heart catheterization with idiopathic PAH, heritable PAH or PAH related to connective tissue disease, repaired congenital systemic to pulmonary shunts, HIV infection, drug use or toxin exposure [16]. Patients had to be in World Health Organization functional class (WHO FC) II–IV, achieve a baseline 6MWD of 50 m or more, and could be either treatment-naïve or receiving a stable dose of phosphodiesterase type 5 inhibitors, oral or inhaled prostanoids, calcium channel blockers, or L-arginine for at least 3 months prior to randomization. Patients receiving intravenous or subcutaneous prostanoids and endothelin receptor antagonists were excluded. After screening, patients were randomized (1:1:1) to placebo, macitentan 3 mg or macitentan 10 mg once daily. Treatment was continued until a primary endpoint event of morbidity/mortality occurred, which indicated the end of double-blind treatment (EOT), or until the end of study (EOS), which was declared when the predefined target of 285 primary endpoint events had occurred, or earlier in the case of premature double-blind treatment discontinuation [16]. Change in 6MWD from baseline to Month 6, time to first occurrence of either PAH-related death or hospitalization up to EOT, and time to all-cause death up to EOS were predefined secondary endpoints of the study. Local institutional review boards or independent ethics committees approved the protocol. A full list of ethics committee/institutional review board(s) that approved the study is provided as supportive information (S1 Table). Written informed consent was obtained from all patients before they entered the study. A special informed consent form was provided to patients who are minors, in accordance with local regulations. A parent or legal representative signed the consent on behalf of a minor. The study was conducted in accordance with the principles of the Declaration of Helsinki.

### Statistical analysis

To evaluate the association between 6MWD and long-term outcome, the risk for PAH-related death or hospitalization up to EOT and for all-cause death up to EOS was calculated among: 1) patients with available 6MWD data at Month 6, dichotomized as above or below the median 6MWD value at Month 6 (400 m), or categorized by quartiles; 2) all randomized patients, categorized according to quartiles of 6MWD values at baseline; and 3) patients with available 6MWD data at baseline and Month 6, categorized according to quartiles of changes in 6MWD at Month 6. Event-free Kaplan–Meier (K-M) estimates of PAH-related death or hospitalization or all-cause death were determined for each categorization of 6MWD values and compared by the log-rank test. Associations were analyzed using univariate Cox regression models and expressed as hazard ratios (HRs) with 95% confidence intervals (CIs). The validity of the proportional hazards assumption was checked in the SERAPHIN study for the primary endpoint of time to morbidity/mortality up to EOT using martingale-based residuals [18].

To account for potential confounding effects, the HRs were also adjusted for study treatment, use of background PAH therapy at baseline and established prognostic markers of long-term outcome in PAH (age, sex, PAH etiology, and WHO FC) [2]. Interaction tests were employed to evaluate potential heterogeneity across the treatment groups (placebo, macitentan 3 mg and macitentan 10 mg) with respect to the associations between 6MWD and long-term outcome. As the *p*-values for interaction indicated no heterogeneity (S2 Table), the associations between 6MWD and long-term outcomes were therefore performed combining all study treatment groups. As the objective was to determine the importance of a second measure of 6MWD on long-term outcome beyond Month 6, only patients with both baseline and Month 6 data were included in the analyses at Month 6. Patients who experienced an event under investigation (PAH-related death or hospitalization, or all-cause death) prior to Month 6 were excluded from these analyses. For the endpoint of PAH-related death or hospitalization up to EOT, patients without an event were right censored at date of premature study drug discontinuation or date of last contact, date of end of study, or date of death for reasons not related to PAH, whichever occurred first. For the endpoint of all-cause death up to EOS, patients without an event were right censored at the end of study date (EOS visit date for completers or date of premature study discontinuation/last contact for patients who did not complete the study as scheduled).

## Results

The 742 patients randomized in the SERAPHIN study were well-matched in terms of demographic, clinical, and disease characteristics at baseline. In total, 76.5% of patients were female, mean (SD) age and time since diagnosis were 45.6 (16.1) years and 2.7 (4.0) years, respectively. Mean (SD) 6MWD was 360 (100.2) meters, approximately half of patients were in WHO FC I/II (52.5%) and WHO FC III/IV (47.5%) and background therapy included phosphodiesterase type 5 inhibitor (61.4%) and oral or inhaled prostanoid therapy (5.4%) [16]. Three patients had missing 6MWD values at baseline.

### Association between 6MWD at Month 6 and long-term outcomes

**Fig 1:**
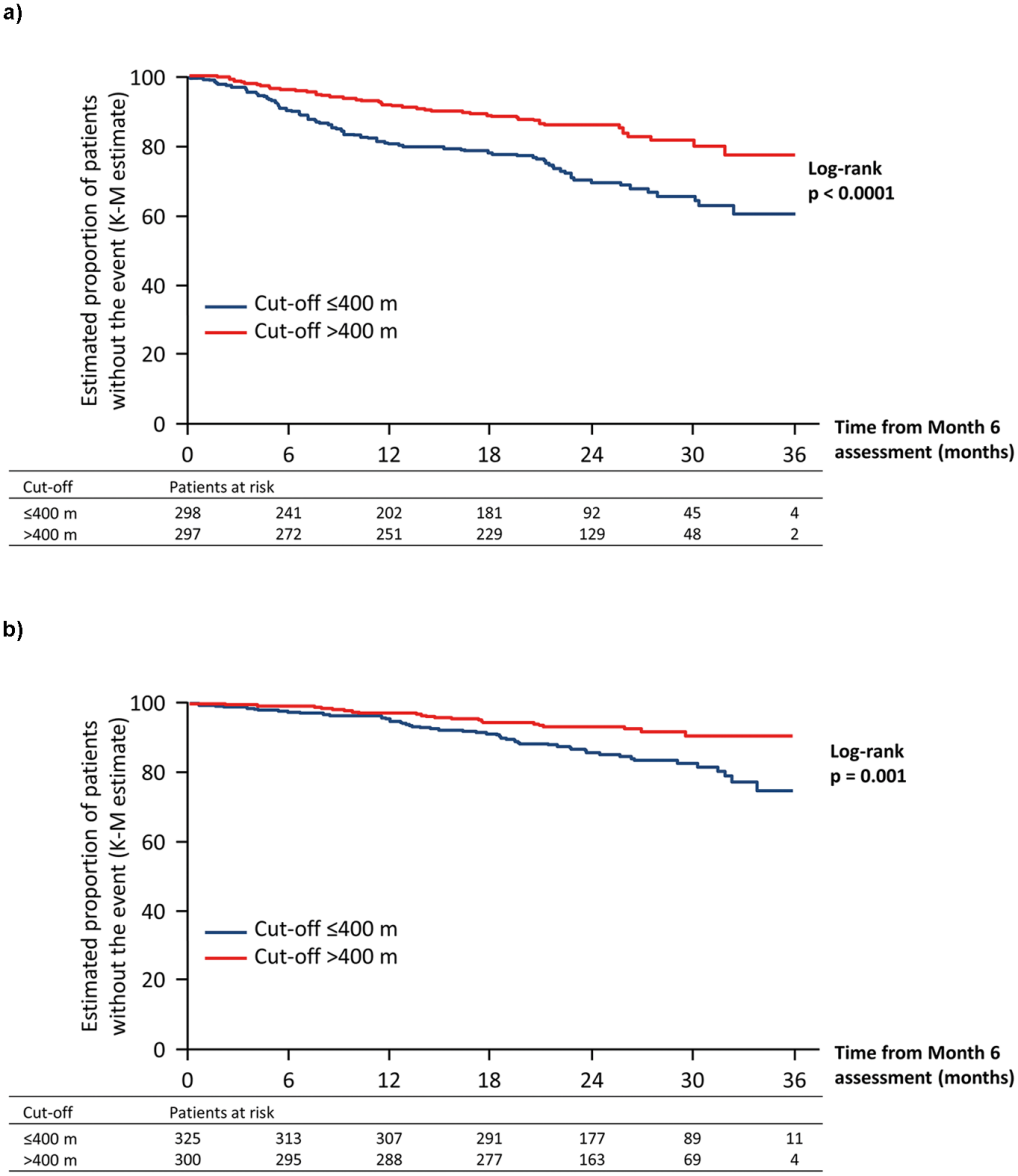
Association between absolute 6MWD applying a cut-off of 400 m at Month 6 and long-term outcomes in patients with PAH: A. PAH-related death or hospitalization up to end of treatment; B. All-cause death up to end of study. 6MWD, six-minute walk distance; K-M, Kaplan–Meier; PAH, pulmonary arterial hypertension.

In the analysis of the risk of PAH-related death or hospitalization, 298 patients had a 6MWD ≤400 m compared with 297 patients with a 6MWD >400 m at Month 6. The number of PAH-related deaths or hospitalization events in these patients was 80 vs. 45, respectively. The risk of experiencing an event was lower among patients with a 6MWD >400 m at Month 6 compared with those with a 6MWD ≤400 m (Fig 1A, Table 1).

**Table 1:** Association between 6MWD and time to PAH-related death or hospitalization up to end of treatment.

6MWD assessment	Categorization	Number of PAH-related deaths or hospitalizations / number of patients	Unadjusted hazard ratio (95% CI) (vs. reference)	Adjusted^a^ hazard ratio (95% CI) (vs. reference)
Month 6	≤400 m; reference	80/298	–	–
	>400 m	45/297	0.48 (0.33–0.69)	0.50 (0.34–0.74)
	Q1 (≤348 m; reference)	49/149	–	–
	Q2 (>348−≤400 m)	31/149	0.55 (0.35–0.87)	0.57 (0.36–0.92)
	Q3 (>400−≤455 m)	25/151	0.40 (0.24–0.64)	0.41 (0.25–0.69)
	Q4 (>455 m)	20/146	0.33 (0.19–0.55)	0.34 (0.20–0.60)
Baseline	Q1 (≤300 m; reference)	81/197	–	–
	Q2 (>300−≤372 m)	53/179	0.56 (0.39–0.79)	0.63 (0.44–0.90)
	Q3 (>372−≤430 m)	40/182	0.39 (0.27–0.57)	0.43 (0.29–0.64)
	Q4 (>430 m)	25/181	0.23 (0.15–0.36)	0.27 (0.16–0.44)
Change from baseline to Month 6	Q1 (≤–9 m; reference)	25/153	–	–
	Q2 (≥–9−≤20m)	32/156	1.02 (0.60–1.72)	1.02 (0.60–1.74)
	Q3 (>20−≤57 m)	29/141	1.01 (0.59–1.73)	0.93 (0.54–1.60)
	Q4 (>57 m)	39/145	1.37 (0.83–2.27)	1.19 (0.71–1.98)

^a^Adjusted for study treatment, use of background PAH therapy at baseline, age, sex, PAH etiology, and WHO FC.

6MWD, six-minute walk distance; CI, confidence interval; PAH, pulmonary arterial hypertension; WHO FC, World Health Organization functional class.

There were 53 deaths among 325 patients who had a 6MWD ≤400 m and 22 deaths among 300 patients who had a 6MWD >400 m at Month 6. Patients who achieved a 6MWD >400 m at Month 6 had a significantly lower risk of all-cause death compared with those who were below this value (Fig 1B, Table 2).

**Table 2:** Association between 6MWD and time to all-cause death up to end of study.

6MWD assessment	Categorization	Number of deaths (all-cause) / number of patients	Unadjusted hazard ratio (95% CI) (vs. reference)	Adjusted^a^ hazard ratio (95% CI) (vs. reference)
Month 6	≤400 m; reference	53/325	–	–
	>400 m	22/300	0.45 (0.27–0.74)	0.45 (0.26–0.78)
	Q1 (≤339 m; reference)	34/157	–	–
	Q2 (>339−≤397 m)	19/158	0.53 (0.30–0.93)	0.49 (0.28–0.89)
	Q3 (>397−≤452 m)	13/154	0.38 (0.20–0.71)	0.34 (0.17–0.68)
	Q4 (>452 m)	9/156	0.27 (0.13–0.55)	0.24 (0.11–0.53)
Baseline	Q1 (≤300 m; reference)	62/197	–	–
	Q2 (>300−≤372 m)	31/179	0.50 (0.33–0.77)	0.56 (0.36–0.88)
	Q3 (>372−≤430 m)	22/182	0.35 (0.21–0.56)	0.36 (0.21–0.60)
	Q4 (>430 m)	11/181	0.18 (0.09–0.34)	0.18 (0.09–0.37)
Change from baseline to Month 6	Q1 (≤–10 m; reference)	16/160	–	–
	Q2 (≥–10−≤19 m)	18/157	1.11 (0.56–2.17)	1.10 (0.56–2.18)
	Q3 (>19−≤55 m)	18/153	1.13 (0.58–2.22)	0.97 (0.49–1.92)
	Q4 (>55 m)	23/155	1.41 (0.75–2.68)	1.19 (0.62–2.29)

^a^Adjusted for study treatment, use of background PAH therapy at baseline, age, sex, PAH etiology, and WHO FC.

6MWD, six-minute walk distance; CI, confidence interval; PAH, pulmonary arterial hypertension; WHO FC, World Health Organization functional class.

**Fig 2:**
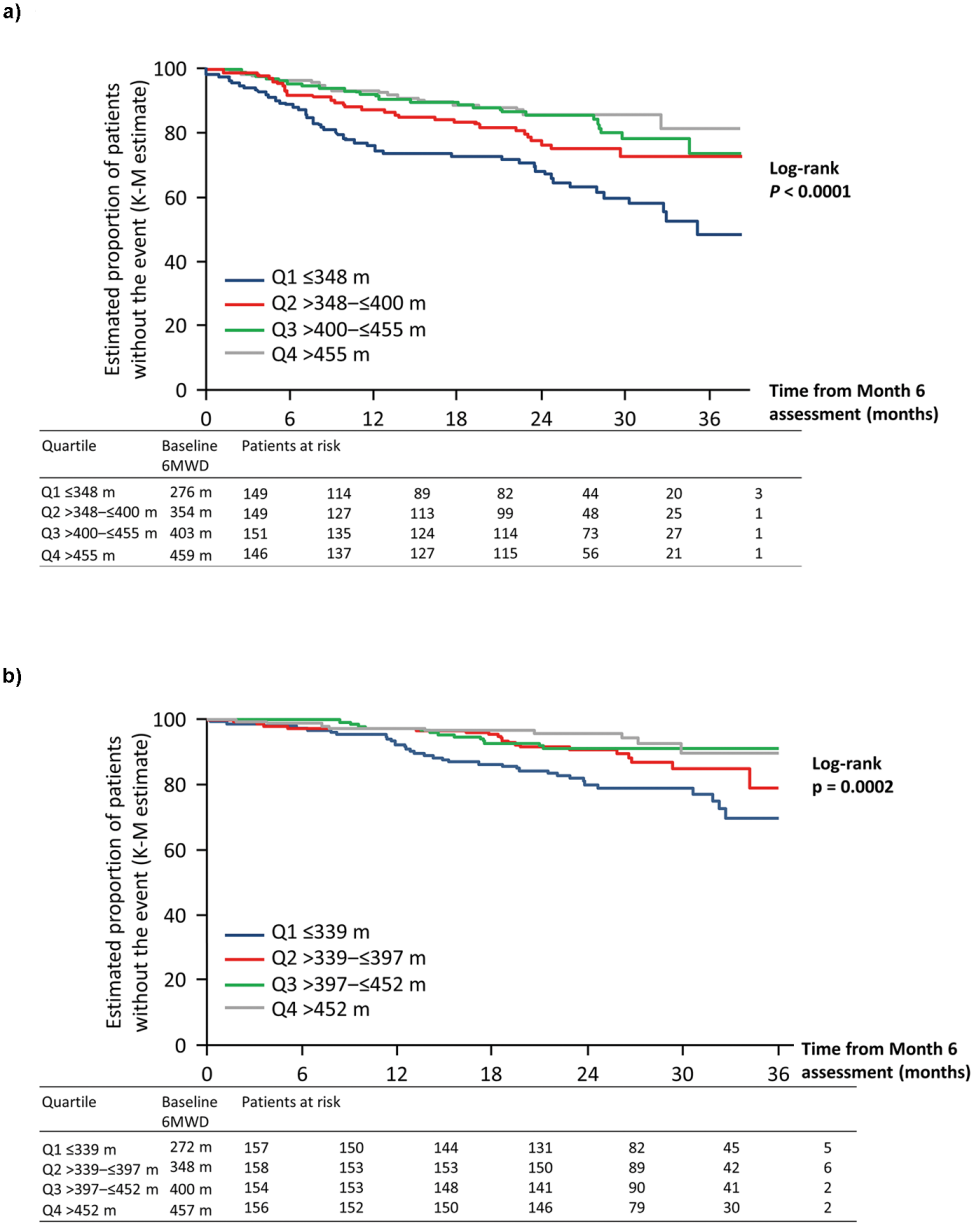
Association between absolute 6MWD quartiles at Month 6 and long-term outcomes in patients with PAH: A. PAH-related death or hospitalization up to end of treatment; B. All-cause death up to end of study. 6MWD, six-minute walk distance; K-M, Kaplan–Meier; PAH, pulmonary arterial hypertension.

Patients with a 6MWD ≤400 m at Month 6 had similar poor long-term outcome whether they started above 400 m (*n* = 28) or below 400 m (*n* = 270) at baseline (adjusted HR 0.95; 95% CI 0.38–2.38). In contrast, among patients with 6MWD >400 m at Month 6, those whose initial baseline 6MWD was below 400 m (*n* = 96) had a trend toward a higher risk of an event compared with those that were above 400 m at baseline (*n* = 201; adjusted HR 1.79; 95% CI 0.98–3.25). Patients in the lowest 6MWD quartile at Month 6 (Q1) had a significantly higher risk of experiencing an event compared with those in the other three quartiles (Fig 2A, Table 1).

The effect of baseline 6MWD quartiles and long-term outcomes at Month 6 was consistent with the observations for PAH-related death or hospitalization. Patients in the lowest 6MWD quartile at Month 6 (Q1) had a significantly higher risk of death compared with those in the other three quartiles (Fig 2B, Table 2).

### Association between 6MWD at baseline and long-term outcomes

**Fig 3:**
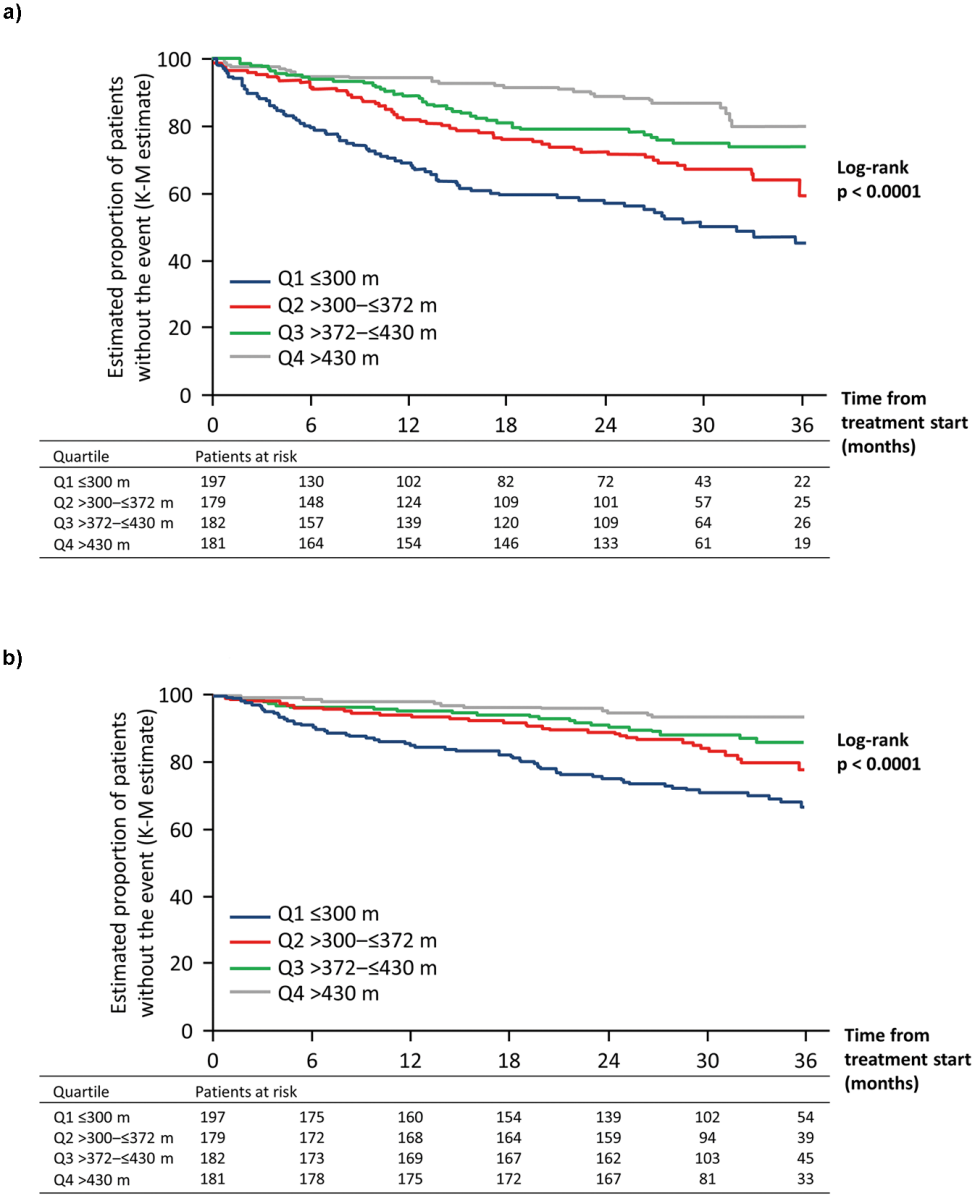
Association between absolute 6MWD quartiles at baseline and long-term outcomes in patients with PAH: A. PAH-related death or hospitalization up to end of treatment; B. All-cause death up to end of study. 6MWD, six-minute walk distance; K-M, Kaplan–Meier; PAH, pulmonary arterial hypertension.

The quartiles of 6MWD measured at baseline in the overall population were: Q1≤300 m (*n* = 197); 300 m<Q2≤372 m (*n* = 179); 372 m<Q3≤430 m (*n* = 182) and Q4>430 m (*n* = 181). In total, 199 patients (26.9%) experienced a PAH-related death or hospitalization event up to EOT (81, 53, 40 and 25 for Q1 to Q4, respectively). Patients in Q1 had a significantly higher risk of experiencing an event compared with those in the other three quartiles (Fig 3A, Table 1).

For all-cause death, 126 (17.1%) events occurred up to EOS (62, 31, 22 and 11 for Q1 to Q4, respectively). Similarly, patients in Q1 had a significantly higher risk of death compared with those in the other three quartiles (Fig 3B, Table 2).

### Association between changes in 6MWD at Month 6 and long-term outcomes

**Fig 4:**
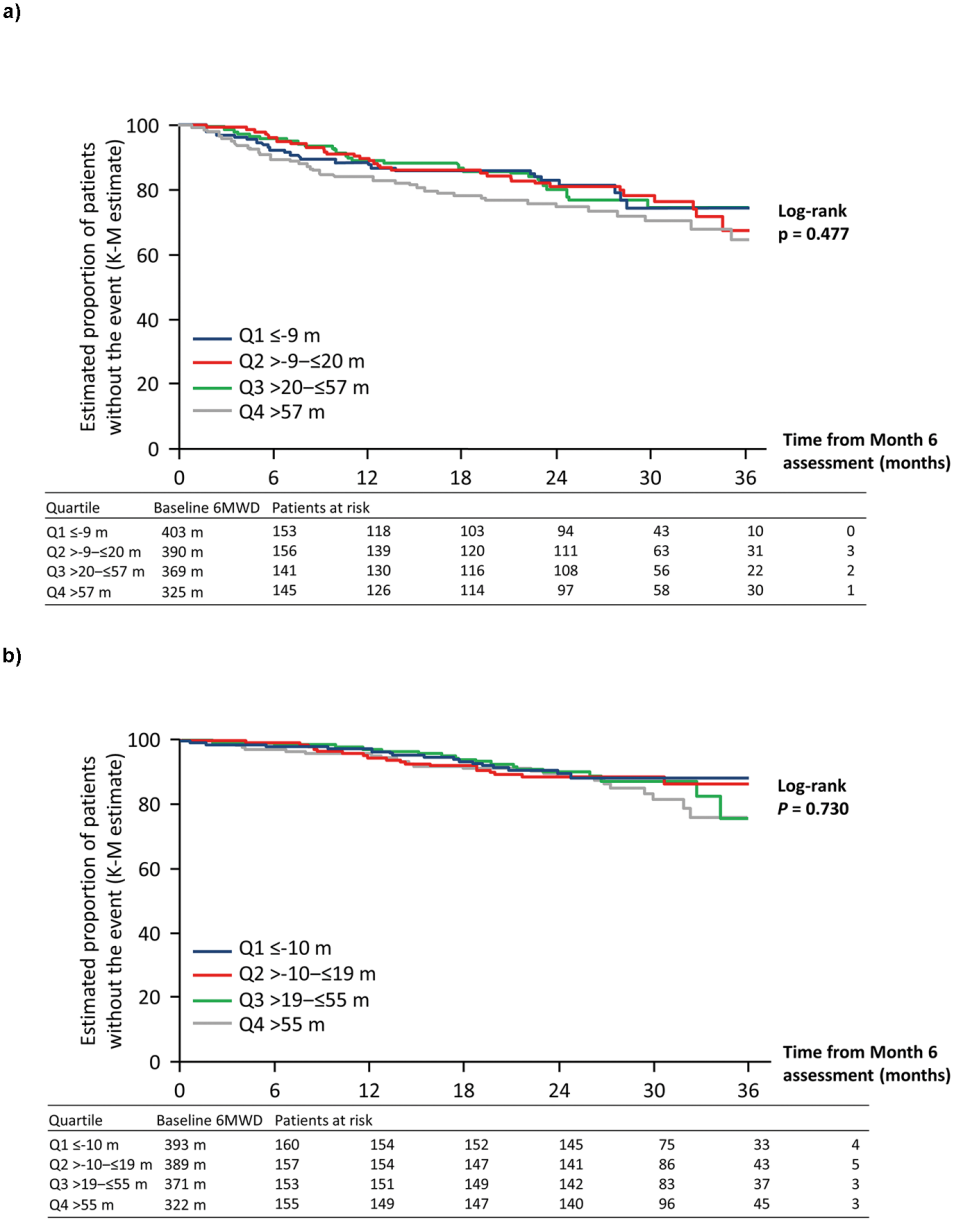
Association between change in 6MWD from baseline to Month 6 (by quartiles) and long-term outcomes in patients with PAH: A. PAH-related death or hospitalization up to end of treatment; B. All-cause death up to end of study. 6MWD, six-minute walk distance; K-M, Kaplan–Meier; PAH, pulmonary arterial hypertension.

There were 595 patients with 6MWD values recorded at Month 6 who did not experience a PAH-related death or hospitalization event prior to Month 6. The quartiles for the change in 6MWD from baseline to Month 6 for these patients were: Q1≤–9 m (*n* = 153); –9 m>Q2≤20 m (*n* = 156); 20 m>Q3≤57 m (*n* = 141), and Q4>57 m (*n* = 145). The greatest changes in 6MWD were observed in patients with the lowest 6MWD at baseline (Fig 4A). The numbers of PAH-related death or hospitalization events that occurred up to EOT were 25, 32, 29 and 39 for Q1 to Q4, respectively. There was no difference in the risk of experiencing an event between the different quartiles (log-rank *p* = 0.477; Fig 4A, Table 1).

For the analysis on all-cause death, there were 625 patients with a 6MWD recorded at Month 6. The quartiles for the change in 6MWD from baseline to Month 6 used to estimate the risk of all-cause death were: Q1≤–10 m (*n* = 160); –10 m>Q2≤19 m (*n* = 157); 19 m>Q3≤55 m (*n* = 153) and Q4>55 m (*n* = 155). Again, the greatest changes in 6MWD were observed in patients with the lowest 6MWD at baseline (Fig 4B). The number of deaths that occurred up to EOS was 16, 18, 18 and 23 for Q1 to Q4, respectively. There was no difference in the risk of all-cause death between the different quartiles (log-rank *p* = 0.730; Fig 4B, Table 2).

## Discussion

SERAPHIN was the first prospective, long-term, randomized controlled study to assess disease progression in more than 700 patients with PAH over a median treatment period of 2.2 years [16]. Patients with a 6MWD of ≥400 m at Month 6 were at reduced risk of future clinical events, such as PAH-related death or hospitalization, and all-cause death. Patients with higher absolute values of 6MWD at baseline or at Month 6 were confirmed as having better prognosis, whereas the magnitude of change in 6MWD during this period was not associated with long-term clinical outcomes.

Since the study of Miyamoto et al. [5], it has been suggested that the evaluation of exercise capacity at baseline, by the 6MWD test, could reflect prognosis in patients with PAH. Other studies provided similar evidence regarding the prognostic value of the 6MWD test [6–8, 17, 19, 20]. Our study confirms that patients with greater 6MWD at baseline have a significantly lower risk of disease progression, supporting the need for assessing 6MWD in daily clinical practice, and that baseline 6MWD represents an important variable to consider in order to better balance clinical study groups in randomized clinical trials.

Absolute 6MWD achieved following PAH treatment has been found to be associated with long-term survival [6, 7, 17, 21]. Sitbon et al. [6], in a study evaluating 178 patients who used intravenous epoprostenol as first-line therapy for PAH, demonstrated that patients who were able to walk ≥380 m after 3 months of PAH-specific therapy had better long-term survival. The same group has shown similar results in patients using first-line monotherapy with bosentan [17]. A more recent study of 2716 patients with PAH enrolled in the US Registry to Evaluate Early and Long-Term PAH Disease Management (REVEAL) found that, among other variables, 6MWD ≥440 m predicted increased survival at 1 year [21]. Based on these findings and on clinical experience, a goal-oriented approach to treatment, which includes a target 6MWD of greater than 380–440 m, is recommended [11, 12]. In the SERAPHIN patient population, when 400 m was applied as a cut-off at Month 6 (corresponding to the median 6MWD at this time point), patients walking <400 m had twice the risk of PAH-related death or hospitalization and of all-cause death compared with those walking more than 400 m. These data confirm the relevance of reassessing 6MWD following initiation of PAH-targeted therapy and the usefulness of establishing absolute thresholds as treatment goals to be pursued in clinical practice [12].

Improving survival would be the ultimate primary endpoint for a disease with high mortality rates. However, studies using survival as the primary endpoint would have a number of caveats, such as the large sample size, long duration of the trial and the use of rescue therapies. In order to reduce sample size and the duration of clinical trials, indirect outcome measures are frequently used [22]. Improvement in 6MWD has been identified as one such indirect outcome measure in PAH and has been used as the primary endpoint in many clinical trials [23, 24]. However, the clinical significance of the observed improvement in 6MWD has become a matter of debate in recent years.

A sub-analysis of the Pulmonary Arterial Hypertension and Response to Tadalafil (PHIRST) trial suggested that increases in 6MWD beyond 33 m would represent the minimal important difference reflecting improved quality of life [25]. However, when looking at the association with mortality, the Ambrisentan in Pulmonary Arterial Hypertension (ARIES) trials indicated that, although an absolute 6MWD after 3 months of treatment was associated with mortality, the change from baseline in 6MWD was not [7]. Furthermore, a meta-analysis of 22 clinical trials in PAH demonstrated that changes in 6MWD did not predict improvements in major clinical events in a short-term follow-up [9]. Another concurrent meta-analysis of ten trials showed that the overall mean 6MWD improvement of 22.4 m accounted for only a small proportion of the observed treatment effect on clinical events [26]. This suggests that change in 6MWD did not represent a valid surrogate endpoint in PAH. Our study confirms that changes in 6MWD after 6 months are not associated with the long-term risk of PAH-related death or hospitalization or with long-term survival. Moreover, patients with the lowest 6MWD at baseline had the greatest increases in 6MWD after 6 months, but still had the worst long-term outcomes. Although exercise capacity improved, the magnitude of the change in 6MWD does not translate into improved long-term outcomes, unless a specific threshold is obtained. These results do not support the use of change in 6MWD at 6 months as a prognostic marker of long-term efficacy of any specific intervention in PAH. Clinically relevant endpoints should be used to demonstrate improvement in long-term outcome.

Our study has limitations that should be acknowledged. This *post hoc* analysis included only a subset of the SERAPHIN population, was not prospectively powered, and the statistical analyses are exploratory in nature and should be interpreted with caution. The baseline characteristics of this subset of the SERAPHIN population were similar to those of the overall SERAPHIN population and balanced between treatment arms. Although our analyses accounted for differences in patient characteristics such as background PAH therapy at baseline, age, sex, PAH etiology and WHO FC, a threshold of 400 m for 6MWD might be a conservative treatment goal for a young patient with idiopathic PAH, or an unachievable goal for an older patient, or for a patient with PAH associated with systemic sclerosis. Nevertheless, it is clear that achieving a functional status threshold, not the magnitude of improvement in exercise capacity, should be the target when developing a treatment strategy.

## Conclusions

In the SERAPHIN study, patients with PAH walking more than 400 m had a lower risk of disease progression, including PAH-related death or hospitalization and all-cause death. Although changes in 6MWD were not associated with long-term outcome in patients with PAH, assessing the absolute 6MWD values remains important in the clinical management of patients with PAH.

## Supporting information

S1 Table List of ethics committee/institutional review board(s) that approved the study. (DOCX)

S2 Table Hazard of PAH-related death or hospitalization and all-cause death events using 6MWD as a continuous parameter. (DOCX)
