# Supplementary material for: Association between six-minute walk distance and long-term outcomes in patients with pulmonary arterial hypertension: Data from the randomized SERAPHIN trial
Source: PLoS One. 2018 Mar 28;13(3):e0193226. doi: 10.1371/journal.pone.0193226 (PMC5873992; doi:10.1371/journal.pone.0193226)
Supplement: S1 Table — (DOCX) [file pone.0193226.s001.docx]

**S1 Table. List of ethics committee/institutional review board(s) that approved the study.**

| **Center** | **Independent Ethics Committee/Institutional Review Board** |
| --- | --- |
| **Argentina** |  |
| 8001 8005 8007 8008 8009 8010 | Comité Independiente de Etica para Ensayos en Farmacologia Clinic |
| 8001 | Comité de Revision Institucional - Sanatoria de la Trinidad Mitr |
| 8002 | Comité de Bioética de la Fundacion Favaloro (Bioethics Comité) |
| 8004 | Comité de Revision Institucional del Hospital Britànico |
| 8005 | Comité de Docencia e Investigacion del Hospital Italiano Garibaldi |
| 8007 | Comité de Docencia e Investigacion del Instituto de Cardiologia Corrientes |
| 8008 | Comité de Revision de Interna del Hospital Privado Centro |
| 8009 | Comité de Etica en Investigaciones Biomedicas - Sanatorio Otamendi y Miroli S.A. |
| 8010 | Comite de Docencia e Investigacion del Hospital Italiano de Cordoba |
| **Australia** |  |
| 5001 | The Alfred Hospital Research & Ethics Unit |
| 5002 | St. Vincent's Hospital Human Research Ethics Committee |
| 5003 | Sydney Local Health District |
| 5004 | Redcliffe-Caboolture Health Service District Ethics Committee |
| **Austria** |  |
| 1001 | Ethikkommission der Medizinischen Universität Wien und des Allgemeinen Krankenhauses der Stadt Wien |
| **Belarus** |  |
| 3101 | Minsk Regional Clinical Hospital |
| 3102 | Vitebsk Regional Clinical Hospital |
| 3104 | LEC of the Republican Scientific-Practical Center "Cardiology" |
| **Belgium** |  |
| 1101 | Faculteit Geneeskunde Commissie voor Medische Ethiek/Klinisch Onderzoek |
| **Bulgaria** |  |
| All sites | Ethics Committee for multi-centric trials |
| **Canada** |  |
| 9001 | Conjoint Health Research Ethics Board - Office of Medical Bioethics |
| 9002 | University Health Network, Research Ethics Board |
| 9002 | Mount Sinai Hospital Research Ethics Board |
| 9003 | University of Western Ontario Office of Research Ethics |
| 9004 | IRB - Comite d'Ethique de la recherche |
| 9005 | University of British Columbia, Clinical Research Ethics Board Office |
| **Chile** |  |
| 8201 | Comite Etica - Científico del Servicio de Salud Metropolitano Central |
| 8201 | Comite de Etica de la Direccion de Investigacion de la Escuela de Medicina de la Pontificia Universidad Catolica de Chile |
| 8202 | Comité de Etica Científico del Servicio de Salud Metropolitano Occidente |
| 8203 | Comitè De Etica Cientifico del Servicio de Salud Metropolitano Oriente |
| **China** |  |
| 5101 | Ethics Committee of Shanghai Pulmonary Hospital |
| 5102, 5103 | Ethics Committee, Renji Hospital, School of Medicine, Shanghai Jiao Tong University |
| 5104 | Ethics Committee of Zhongshan Hospital Fudan University |
| 5105 | Ethic Committee of Peking Union Medical College Hospital for Clinical Trials |
| 5106 | IEC of Beijing Anzhen Hospital of the Capital University of Medical Sciences |
| 5107 | IEC of Chinese PLA General Hospital |
| 5108 | Ethic Committee of Guangdong General Hospital |
| 5109 | IEC of The First Affiliated Hospital with Nanjing Medical University |
| **Colombia** |  |
| 8302 | Comité de Ética e Investigaciones Fundación Clínica Shaio |
| 8303 | Comite de Etica en Investigacion Fundacion Cardiovascular de Colombia |
| **Croatia** |  |
| 3002 | Središnje etičko povjerenstvo, Agencija za lijekove i medicinske proizvode |
| **Denmark** |  |
| 1201 | Den Videnskabsetiske Komité for Region Midtjylland |
| **Finland** |  |
| All sites | Etiska Kommittéerna |
| **France** |  |
| All sites | CPP Ile-de-France VII |
| **Germany** |  |
| All sites - 1401 | Ethik-Kommission Fachbereich Medizin, Justus-Liebig- Universität |
| 1403 | Ethikkommission an der Medizinischen Fakultät Leipzig Institut für klinische Pharmakologie |
| 1404 | Landesamt für Gesundheit und Soziales, Geschäftsstelle der Ethik-Kommission des Landes Berlin |
| 1406 | Ethik-Kommission der Medizinischen Fakultät der Universität zu Köln |
| 1408 | Ethikkommission Aerztekammer Hamburg, Körperschaft des Öffentlichen Rechts |
| 1409 | Ethikkommission der Medizinischen Fakultät der TU Dresden |
| 1410 | Ethikkommission an der Medizinischen Fakultät, Ernst-Moritz-Arndt-Universität Greifswald, Institut für Pharmakologie |
| 1411 | Ethik-Kommission der Medizinische Fakultät der Ludwigs-Maximilians Universität |
| 1412 | Ethikkommission der Medizinischen Fakultät Heidelberg |
| 1413 | Ethikkommission der Medizinischen Fakultät der Universität Regensburg |
| 1415 | Ethik-Kommission bei der Ärztekammer des Saarlandes |
| **Hong Kong** |  |
| 5201 | Institutional Review Board of the University of Hong Kong / Hospital Authority Hong Kong West Cluster |
| 5202 | Chinese University of Hong Kong - New Territories Ethics Committee |
| **Hungary** |  |
| All sites | Egészségügyi Tudományos Tanács Klinikai Farmakológiai Etikai Bizottság (Medical Research Council Ethics Committee for Clinical Pharmacology) |
| 3401 | Szegedi Tudományegyetem, Szent-Györgyi Albert Klinikai Központ Regionális és Intézményi Humán Orvosbiológiai Kutatásetikai Bizottsága |
| 3404 | University of Pécs, Faculty of Medicine, Medical Microbiology and Immunity Institute, Regional Research Ethics Committee of the Medical Center |
| 3405 | Gottsegen György Orszagos Kardiologiai Intézet Etikai Bizottsaga |
| 3406 | Semmelweis Egyetem Intézeti Tudomanyos és Kutatásetikai Bizottság |
| **India** |  |
| 5301 | Ethics Committee Apollo Hospitals International Ltd |
| 5302 | Institutional Ethics Committee- Deenanath Mangeshkar Hospital & Research Centre |
| 5303 | Clinical Research & Ethics Committee, National Health & Education Society |
| 5304 | Institutional Ethics Committee, Care Foundation |
| 5305 | Ethical Research Committee-Narayana Hrudayalaya Institute of Medical Sciences |
| 5306 | Institutional Ethics Committee, Maulana Azad Medical College, New Dehli |
| 5307 | Ethics Committee For Research On Human Subjects |
| **Israel** |  |
| 7101 | Helsinki Committee Carmel Medical Center |
| 7102 | Helsinki Committee Rabin Medical Center |
| 7103 | Prof. Amichai Rubin / Prof. Moshe Berant |
| 7104 | The Helsinki Commitee Chaim Sheba Medical Center |
| 7105 | Helsinki Committee Kaplan Medical Center |
| 7106 | The Helsinki Committee Tel Aviv Sourasky Medical Center |
| **Italy** |  |
| 1501 | Comitato Etico dell'Azienda Policlinico Umberto I di Roma - c/o Servizio Amministrativo Area Scientifica e di Ricerca |
| 1502 | Fondazione IRCCS, Policlinico "San Matteo" Comitato de Bioetico |
| **Malaysia** |  |
| 5401 | Ethics Comittee Institut Jantung Negara |
| **Mexico** |  |
| 8401 | Ethics Commitee Instituto Nacional de Cardiologia Ignacio Chavez Comision de Investigacion del Instituo Nacional de Cardiologia Igancio Chavez |
| 8402 | Comité de Ética e Investigación de la Unidad de Investigación Clínica en Medicina. |
| **Netherlands** |  |
| All sites | Verenigde Commissies Mensgebonden Onderzoek |
| **Norway** |  |
| 1701 | Regional komité for medisinsk og helsefaglig forskningsetikk, Sør-Øst-Norge |
| **Peru** |  |
| All sites | Comite Institucional de Etica en Investigacion de la Universidad de San Martin de Porres - Clinical cada mujer |
| 8501 | EC Facultad de Medicina Humana Uni de San Martin de Porres |
| 8502 | Comision Reguladora De Estudios Clinicos Del Completo Hospitalario San Pablo |
| 8503 | Comite de Bioetica De La Red Asistencial Sabogal-Essalud Hospital Nacional Alberto Sabogal Sologuren, Essalud |
| **Poland** |  |
| All sites | Komisja ds. Etyki Badań Naukowych przy Instytucie Gruźlicy i Chorób Płuc |
| **Romania** |  |
| All sites | Comisia Nationala de Etica pentru Studiu Clinic al Medicamentului |
| **Russian Federation** |  |
| All sites | Ethics Council at Minzdravsocrazvitiya |
| 3801 | The Joint Local Ethics Committee of the Municipal Budgetary Healthcare Institution “Kemerovo Cardiology Dispensary” |
| 3802 | Committee on the Ethics issues in clinical cardiology of the Federal State Institution “Russian Cardiology Scientific and Production complex of Minzdravsotsrazvitiya of Russia” |
| 3803 | Biomedical Ethics Committee at Federal State Budgetary Institution “Scientific Research Institute of Cardiology” Siberian branch of RAMS |
| 3804 | Ethics Committee of the Regional Budgetary State Health Care Institution “Tomsk Regional Clinical Hospital” |
| 3805 | Committee on the Ethics of clinical trials of Sverdlovsk Regional Clinical Hospital #1 |
| 3806 | Ethics Committee at the “Scientific Research Institute of Pulmonology” of Federal Medical and Biological Agency of Russia |
| 3807 | Ethics Committee of the Municipal Health Care Institution of Yaroslavl Region "Clinical Hospital of Emergency Care named after N.V. Solovyov" |
| 3808 | Local Independent Ethic Committee of National Research Centre for Preventive Medicine |
| 3809 | Ethics committee on clinical trials of the City Clinical Hospital #1 N.I. Pirogov |
| 3810 | Ethics committee of St. Petersburg Medical Academy of Postgraduate Education |
| 3811 | Ethics Committee at the Federal State Budgetary Institution “Federal Center of heart, blood and endocrinology named after V.A.Almazov” of the Ministry of Health Care and Social Development of the Russian Federation |
| 3812 | Ethics Committee of St. Petersburg State Medical University n.a. Pavlov |
| **Serbia** |  |
| 3901 | Univerzitetska dečja klinika, Etički odbor |
| 3902 | Kliničko Bolnički Centar Zemun, Etički Komitet |
| 3903 | Klinički Centar Srbije, Etički Odbor |
| **Singapore** |  |
| 5501 | Singapore General Hospital Ethics Committee |
| 5501 | Singapore Health Services Pte Ltd. Domain C |
| 5502 | National Healthcare Group Domain Specific Review Board, Domain C |
| **Slovakia** |  |
| All sites | Etická komisia NÚSCH |
| **South Africa** |  |
| 6001, 6002 | University of the Witwatersrand Human Research Ethics Committee |
| 6003 | Pharma Ethics (Pty) Ltd |
| 6004 | University of Stellenbosch Committee for Clinical Trials |
| **Spain** |  |
| All sites | Hospital Vall d' Hebrón, Fundacio per la Recerca |
| 1902 | Comité Etico de Investigacion Clinica |
| 1904 | Comité Etico de Investigacion Clinica |
| 1907 | Comité Etico de Investigacion Clinica |
| **Sweden** |  |
| All sites | Regionala Etikprövningsnämnden i Uppsala |
| **Taiwan** |  |
| 5601 | Research Ethics Committee National Taiwan University Hospital |
| 5602 | The Institutional Review Board of Taichung Veterans General Hospital |
| 5602 - central | Joint Institutional Review Board |
| **Thailand** |  |
| 5701 | Ethical Clearance Committee on Human Rights Related to Researches Involving Human Subjects Faculty of Medicine, Ramathibodi Hospital, Mahidol University |
| 5702 | Ethics Committee Faculty of Medicine Siriraj Hospital, Mahidol University |
| 5703 | Research Ethics Committee, Faculty of Medicine, Chiang Mai University |
| 5704 | The Khon Kaen University Ethics Committee for Human Research |
| **Turkey** |  |
| All sites | Hacettepe Üniversitesi Tip Fakültesi Tibbi, Cerrahi ve Ilac Arastirmalari Etik Kurulu |
| **Ukraine** |  |
| All sites | Central Ethics Committee of the Health Ministry of Ukraine |
| 4101 | Bioethics Committee affiliated to Dnepropetrovsk State Medical Academy |
| 4102 | Local Ethic Committee of Lviv Regional Clinical Hospital |
| **United Kingdom** |  |
| 2101 | London – Hampstead (Formerly: EC Royal Free Hospital & Medical School Research Ethics Committee) |
| **USA** |  |
| 9101 | Colorado Multiple IRB |
| 9102 | IRB - Columbia University Medical Center |
| 9103 | IRB - Western |
| 9104 | IRB - Western |
| 9105 | IRB - Mayo Clinic Foundation |
| 9106 | IRB - Western |
| 9107 | IRB - Louisiana State University Health Sciences Center |
| 9108 | IRB - Intermountain Healthcare Urban Central Region |
| 9109 | IRB - Western |
| 9110 | IRB - Boston University Medical Center |
| 9111 | IRB - Western |
| 9112 | IRB - Western |
| 9114 | IRB - Western |
| 9115 | IRB - The University of Michigan Medical School |
| 9116 | IRB - Western |
| 9118 | IRB - Western |
| 9119 | IRB - UTHSC-Committee for the Protection of Human Subjects |
| 9121 | IRB - Wayne State University - Human Investigation Committee |
| 9123 | IRB - University of California San Diego Human Research Protections Program |
| 9125 | IRB - University of Kansas Medical Center - Human Subjects Committee |
| 9126 | IRB - Washington University School of Medicine - Human Research Protect. Office |
| 9127 | IRB - Western |
| 9128 | IRB - Baylor College of Medicine |
| 9129 | IRB - The University of Chicago - Section of Regulatory Compliance |
| 9130 | IRB - University of Maryland School of Medicine - Human Research Protection Office |
| 9131 | IRB - Cottage Health System |
| 9132 | IRB - Western |
| 9133 | IRB - Duke |
| 9134 | IRB - Partners Human Research Committee (Boston) |
| 9135 | IRB - Maine Medical Center - Office of Research Affairs |
| 9136 | IRB - Aurora |
| 9137 | IRB - Western |
| 9138 | IRB - Vanderbilt University |
| 9139 | IRB - Veterans Affairs Greater Los Angeles Healthcare System - 151 |
| 9140 | IRB - University of Texas Southwestern Medical Center at Dallas |
| 9142 | IRB - Allegheny General Hosp |
| 9143 | IRB - Western |

IEC, Independent Ethics Committee; IRB, Institutional Review Board
