# Supplementary material for: Association between six-minute walk distance and long-term outcomes in patients with pulmonary arterial hypertension: Data from the randomized SERAPHIN trial
Source: PLoS One. 2018 Mar 28;13(3):e0193226. doi: 10.1371/journal.pone.0193226 (PMC5873992; doi:10.1371/journal.pone.0193226)
Supplement: S2 Table — (DOCX) [file pone.0193226.s002.docx]

**Supporting Information**

**S2 Table. Hazard of PAH-related death or hospitalization and all-cause death events using 6MWD as a continuous parameter.**

| **Endpoint** | **6MWD assessment** | **Treatment arm** | **Hazard ratio**  **(95% CI)** | **Interaction**  ***p*-value** |
| --- | --- | --- | --- | --- |
| PAH-related death or hospitalization up to EOT | Baseline | Macitentan 10 mg | 0.52 (0.38,0.70)^a^ | 0.297 |
|  |  | Macitentan 3 mg | 0.67 (0.53,0.84)^a^ |  |
|  |  | Placebo | 0.54 (0.45,0.66)^a^ |  |
|  | Month 6 | Macitentan 10 mg | 0.48 (0.32,0.74)^a^ | 0.114 |
|  |  | Macitentan 3 mg | 0.80 (0.57,1.13)^a^ |  |
|  |  | Placebo | 0.54 (0.42,0.70)^a^ |  |
|  | Change from baseline  to Month 6 | Macitentan 10 mg | 1.06 (0.95,1.18)^b^ | 0.157 |
|  |  | Macitentan 3 mg | 1.07 (0.97,1.17)^b^ |  |
|  |  | Placebo | 0.94 (0.84,1.04)^b^ |  |
| All-cause death up to EOS | Baseline | Macitentan 10 mg | 0.42 (0.30,0.60)^a^ | 0.157 |
|  |  | Macitentan 3 mg | 0.65 (0.50,0.86)^a^ |  |
|  |  | Placebo | 0.53 (0.40,0.69)^a^ |  |
|  | Month 6 | Macitentan 10 mg | 0.54 (0.38,0.78)^a^ | 0.391 |
|  |  | Macitentan 3 mg | 0.76 (0.54,1.07)^a^ |  |
|  |  | Placebo | 0.63 (0.43,0.93)^a^ |  |
|  | Change from baseline  to Month 6 | Macitentan 10 mg | 1.07 (0.94,1.21)^b^ | 0.550 |
|  |  | Macitentan 3 mg | 1.00 (0.90,1.11)^b^ |  |
|  |  | Placebo | 1.08 (0.96,1.22)^b^ |  |

^a^Per every 100 m more in 6MWD.

^b^Per every 20 m increase in 6MWD.

6MWD, six-minute walk distance; CI, confidence interval; EOS, end of study; EOT, end of treatment; PAH, pulmonary arterial hypertension.
